# Infectious Disease, Endangerment, and Extinction

**DOI:** 10.1155/2013/571939

**Published:** 2013-01-16

**Authors:** Ross D. E. MacPhee, Alex D. Greenwood

**Affiliations:** ^1^Vertebrate Zoology, American Museum of Natural History, Central Park West at 79th Street, New York, NY 10024, USA; ^2^Leibniz-Institute for Zoo and Wildlife Research, Department of Wildlife Diseases, Alfred-Kowalke-Straße 30, 10315 Berlin, Germany

## Abstract

Infectious disease, especially virulent infectious disease, is commonly regarded as a cause of fluctuation or decline in biological populations. However, it is not generally considered as a primary factor in causing the actual endangerment or extinction of species. We review here the known historical examples in which disease has, or has been assumed to have had, a major deleterious impact on animal species, including extinction, and highlight some recent cases in which disease is the chief suspect in causing the outright endangerment of particular species. We conclude that the role of disease in historical extinctions at the population or species level may have been underestimated. Recent methodological breakthroughs may lead to a better understanding of the past and present roles of infectious disease in influencing population fitness and other parameters.

## 1. Background

Although lethal epi- or panzootics are obvious risk factors that can lead to population fluctuation or decline in particular circumstances, infectious diseases are seldom considered as potential drivers of extirpation or extinction—that is, of the complete loss of all populations or subunits comprising a given biological species. For example, in conservation biology, infectious disease is usually regarded as having only a marginal or contributory influence on extinction, except perhaps in unusual circumstances (e.g., [1–4]). In their examination of 223 instances of critically endangered species listed by the IUCN (International Union for Conservation of Nature) as allegedly threatened by infectious disease, Smith et al. [4] found that in the overwhelming majority of cases there was no conclusive evidence to support infectious disease as a contributing threat. Although this record should improve with increasing awareness of the effects of infectious diseases on wildlife, as this paper illustrates progress has so far been slow.

 Both of the authors of this paper are primarily concerned with mammals, which is the group that will receive the bulk of attention here. However, at the pragmatic, data-gathering level, the issues concerned with properly accounting for and evaluating the effects of infectious diseases on natural populations differ little from one phylogenetic grouping to another. 

First, narrowing down extinction events or even catastrophic population declines to single causes is almost always problematic. In most real cases, extinction is multicausational, even if one cause can be identified as being predominantly responsible [5]. Habitat fragmentation and climate change are currently regarded as the leading prime movers behind most instances of extreme endangerment, to which other stressors such as pollution, invasive competitors, and so forth, might be of greater or lesser importance in particular circumstances. Disease, however, is rarely mentioned as a possible contributing factor in such contexts (but see [6]).

Another difficulty is lack of knowledge about pathogen diversity and susceptibility in wildlife. In the absence of sufficient means of detection and characterization, it is difficult to assess or to give quantitative expression to the degree to which pathogens might influence population decline or extinction. Thus it has been estimated that only a small fraction of bacterial diversity has been identified at even the most basic systematic level. This problem is exacerbated in the case of viruses, which often evolve rapidly and defy, in any case, classical methodologies for identifying “species” [7]. For example, bat viruses have only recently begun to be described systematically, even though many chiropterans are known vectors of numerous zoonotic diseases and corporately represent the second largest grouping (by species richness) of mammals after rodents [8, 9]. A similar lack of knowledge affects our understanding of parasites and fungi that affect wildlife. 

The foregoing difficulties are compounded when one considers that, unless a species is studied extensively during and up to the actual extinction event affecting it, all extinction studies are retrospective. Retrospective investigation of losses in which disease is possibly implicated is often severely hindered by limitations in the number and quality of samples available for study, as well as the inability to satisfy Koch's postulates—especially if both host and pathogen became extinct simultaneously [10]. Performing isolation, reisolation, and reinfection experiments to directly establish that a particular pathogen was indeed the causative agent behind a given infection is either very difficult or impossible to do retrospectively. Isolation and recreation of the 1918 H1N1 influenza A virus [11], for example, were performed by sequencing from extractions derived from individuals thought to have died of the disease in WWI, not by directly isolating the infectious virus from tissues (as would be required to formally comply with Koch's postulates). Although most studies will have to be correlative rather than dispositive, one can nevertheless test hypotheses concerning plausible causal agents and examine samples for presence/absence of specific pathogens [12]. 

Forensically, decay, degradation, and chemical changes in DNA post mortem produce severe methodological challenges to retrieving and accurately determining sequences [13]. In addition, in any retrospective investigation involving “ancient DNA,” pathogen nucleic acids will be less abundant than those of the host, and this dilution effect will make sequence retrieval even more complex [10]. For example, relatively abundant mitochondrial DNA is generally easier to retrieve from fossils or historical samples than lower copy per cell nuclear DNA. Pathogen nucleic acids are generally even lower copy than host DNA sequences in a given extraction. These and other factors reviewed here may help to explain the paucity of conclusive studies of disease-mediated extinction, except in the very few instances in which sampling and methodological roadblocks could be overcome. Nonetheless, in favorable circumstances it should be possible to genetically analyze ancient pathogens with sufficient accuracy to make the endeavor worthwhile, especially because next-generation sequencing methods are beginning to make such endeavors ever more feasible [14–18].

Why should the possible role of infectious disease in endangerment and extinction be regarded as a critical issue in modern conservation? Whether or not disease was ever a major cause of extinction in the fossil record [19], in our times it plays an acknowledged but perhaps underestimated role. Pathogen-driven population declines have been identified in a wide array of invertebrate and vertebrate taxa (cf. [20]), suggesting that the phenomenon is probably universal. Yet without the kinds of monitoring methods now available, some and perhaps most of these declines would have gone undetected, or attributed to other causes. Further, the processes forcing such declines are as diverse as the pathogens themselves and are far from being clearly understood. The apparent increase in zoonotic diseases during the last few decades [21] may be objectively real or merely due to better monitoring, but it seems highly likely that loss or reduction of pristine habitats and the overall impact of invasive species should promote the introduction of opportunistic pathogens into wildlife with increasing frequency. 

Thus, understanding the dynamics of disease-mediated species declines may be critical to conservation missions concerned with a wide variety of species and habitats. Recent advances in molecular biology and microbiology have permitted the detection and identification of hosts of novel microorganisms, many of which are pathogenic, and the technology needed to assess threat levels is becoming increasingly available.

## 2. Disease as an Agent of Extinction: Some Considerations

Although the fossil record clearly establishes that the fate of all species is to eventually die out, it is obvious from the same record that the rate of disappearance of individual species varies significantly [22]. As already noted, inferences about how (as opposed to when) an individual species disappeared must be developed inductively and retrospectively. An important guideline is that apparent causes of extinction that are diachronic (repeatedly affect species across time) are inherently more plausible than ones that are claimed to have occurred only once, or apply to only one taxon. Although this means that explanations about individual extinctions are not strictly testable, they can nevertheless be evaluated in terms of likelihood, which is the approach currently taken by the International Union for Conservation of Nature (IUCN) and several other conservation organizations interested in compiling extinction statistics [23, 24]. 

It is an accepted tenet in conservation biology that any severe, continuing threat to a species might eventually contribute to its extinction [25]. From this perspective, it is also accepted that diseases presenting with very high levels of mortality—as in the case of a highly transmissible infection that is newly emergent in a population—can cause outright endangerment. But are there conditions under which a disease, probably in combination with other threats, might so imperil a species to cause its complete disappearance? MacPhee and Marx [19] considered this issue from the standpoint of model pathogenic features that a disease-provoking organism might exhibit in forcing the extinction of a given species. These features include:

1. a reservoir species presenting a stable carrier state for the pathogen,
2. a high potential for causing infections in susceptible species, affecting critical age groups,
3. a capacity for hyperlethality, defined here as mortality rates in the range of 50–75%.

Only under the most extreme conditions is it conceivable that a species would suffer extinction in a single epizootic event. Much more likely would be repeated outbreaks over a period of years gradually reducing the fitness level of the species, with final disappearance potentially caused by stochastic events (such as causally unassociated climate change). One way in which this condition might be achieved would be through a stable carrier (i.e., a species other than the target, living in similar circumstances in the same environment, and in which the infection is inapparent or at least sublethal). A well-studied example is the transfer of simian acquired immunodeficiency virus from one species of macaque to another [26]. Although this instance occurred under captive conditions, repeated outbreaks of distemper in lions and African wild dogs have long been thought to be due to transfer from domestic dogs (although the mechanism is debated; see [27]). Obviously, for a disease to have a very severe impact, it would be necessary for the pathogen to occur in highly lethal, aggressive strains that strongly impact the target species before attenuated strains arise and become common.

High potential for causing infections in a susceptible species is usually associated with the ability to successfully enter the organism through a major portal, such as the respiratory tract, where it can be lodged and transmitted easily (e.g., via aerosol). To achieve hyperlethality and produce serious mortality, all age groups within a species would probably have to be susceptible, not just the very young or very old (or the immunocompromised), with death the usual outcome. In large-bodied mammals, a fundamental consideration is that any process that deleteriously affects young individuals will have a pronounced effect on survivorship because of the lengthy intervals in birth spacing [19].

Lethality in the range of 50–75% is obviously extremely high and thus extremely unusual, although historically seen in Ebola infections in humans and in experimental transmission studies from pigs to macaques [28]. High percentages may have also been achieved in rinderpest outbreaks among East African bovids in the early 20th century [29], although quantitative data on this are largely lacking. An important issue here, however, is whether pathogens causing this level of lethality could maintain themselves in nature long enough to seriously imperil a species. Speculatively, a possible outcome with hyperlethal infections producing a rapid, fatal outcome is that affected populations would be reduced to small numbers of widely dispersed and/or relatively or completely immune individuals. Under these circumstances, the epizootic would necessarily abate as it ran out of new hosts, leading to the conclusion that exceptionally lethal diseases cannot be indefinitely maintained in a population or species under normal circumstances. However, if reservoirs exist from which the pathogen could repeatedly emerge, in principle epizootics might resurge year after year until population sizes were reduced below viable levels (~50–500 individuals). At this point stochastic effects might intervene and lead to complete loss of the species. Among possible examples of this “perfect storm” of circumstances and consequences is the loss of Christmas Island rats, detailed elsewhere in this paper. Among birds, the severe impact of avian malaria on Hawaiian honeycreepers is also pertinent and discussed later in this paper. Although a number of honeycreeper species survive at high elevations, above the limit at which introduced *Culex* mosquitos can survive, there are multiple adventitious threats, such as deforestation and competition from invasive species, which add to their endangerment picture [30].

## 3. Extinction and Infectious Disease in Invertebrates 

Demonstrating that disease can produce endangerment and even extinction in species of invertebrates is not inherently more difficult than demonstrating the same thing for vertebrates. However, because there tend to be far fewer specialists for individual invertebrate groups, save for those having some degree of economic significance, the chances are high that disease impacts will frequently be missed. One case that was not missed was the loss of the last members of *Partula turgida*, a snail from French Polynesia that succumbed to an infection of the microsporidian *Steinhausia* sp. [31]. *Steinhausia* is a known parasite of various taxa of bivalve molluscans, infecting oocytes and thereby reducing fecundity (e.g., “mussel egg disease”; [32]). The decline of *P. turgida* received an unusual level of attention (for an invertebrate) because of this species' importance for studying evolutionary variation and niche occupation [33]. This snail and several of its close relatives were already considered extinct in the wild due to predation by the introduced wolfsnail *Euglandina* when the last few known individuals were collected and kept as a captive colony. As is frequently the case with unmanaged natural populations, there were no relevant baseline data for this species, and it cannot be excluded that *Steinhausia* was already present in the colony. Admittedly, so small a coterie of individuals hardly constituted a viable species and could have been driven to extinction by other mechanisms as well. It should also be noted that *Steinhausia* infections are not known to present a severe threat to any natural populations of bivalves, or at least any that have maintained normal populations. 

Other possible instances of extinction by disease in invertebrates are few and inconclusive [1], although the loss of the eelgrass limpet (*Lottia alveus*) may be mentioned in this context as it demonstrates that “pathogen pollution” can have severe *indirect* effects on species other than the one affected by disease [34]. In this case, eelgrass wasting disease killed off seagrasses on both sides of the Atlantic on such a massive scale that all known populations of this limpet terminally crashed [35].

## 4. Extinction and Infectious Disease in Amphibians 

In their survey Smith et al. [4] noted that amphibians account for 30% of critically endangered animals and that they also comprise approximately 75% of critically endangered species threatened by disease. Although ranavirus infections, trematode infestations, and several other pathogen threats have been proposed as driving particular cases of decline, the agent thought to contribute most widely to amphibian endangerment is *Batrachochytrium dendrobatidis*, first identified in the 1990s as the cause of a fatal chytridiomycosis [36, 37]. 

A concern that has emerged in the last few years is the effect of the global trade in amphibians on the health of natural populations. There are several instances of ranavirus and chytrid infections having been detected in pet store populations [38]; if infected individuals were to escape, transmission to wildlife is an obvious possibility. Release of captive-bred animals in reintroduction experiments represent another potential danger if captives were exposed to virulent pathogens. Amphibians are under global assault from a large variety of impactors additional to infectious diseases, including habitat loss, pollution, pesticides, and harmful UV radiation. Perhaps as many as 120 amphibian species have already been driven to extinction in recent decades; however, because of the complexity of the factors affecting their endangerment, the number actually lost to the effects of infectious disease remains uncertain [4, 39].

## 5. Extinction and Infectious Disease in Birds

Smith et al. [4] identified 18 examples of bird extinctions and extirpations that have been attributed at least partly to infectious diseases. Of these, 16 cases concern endemics that lived in the Hawaiian Islands; most were from one tribe of finches, the Hawaiian honeycreepers (Drepanidini, Fringillidae). Warner [40] proposed that these losses were due to panzootics caused by the inadvertent introduction of *Culex quinquefasciatus*, a vector of avian malaria (*Plasmodium relictum*). Another lethal agent, presumably introduced as well, was avian pox (*Poxvirus avium*). 

Although there are no empirical observations relating to the hypothesized panzootics (which presumably occurred in the early 19th century, when the mosquito arrived), one obvious effect of massive population depletions was that surviving species of honeycreepers—formerly common in the lowlands—became restricted to higher elevations where the mosquitoes do not occur. Additional research in more recent years indicates that such diseases limit the distribution and abundance of susceptible species, and that it is these factors (grouped as habitat loss) that primarily govern local or complete extinction [41, 42].

## 6. Extinction and Infectious Disease in Mammals

Although there are several current examples of mammalian species already under threat for various reasons being severely impacted by infectious diseases—including canine distemper in black-footed ferrets and lions [43, 44], Ebola and Marburg hemorrhagic diseases in anthropoids [45], and transmissible facial tumour disease in Tasmanian devils [46]—none of these has (yet) resulted in extinction. Possible examples in the mammalian fossil record (cf. [19]), although compelling in some instances, lack adequate corroboration for reasons already discussed. Indeed, to date there is only one study [10] that may be said to meet appropriate retrospective criteria for identifying disease as the primary cause of extinction at the species level in any mammal. This study involved an investigation of the disappearance of two endemic murid species on Christmas Island (Indian Ocean) at the beginning of the 20th century. 

That the Christmas Island extinctions could be usefully studied at all is due in large measure to the work of the biologist Andrews [47] and, later, the parasitologist Durham [48], both of whom spent considerable time on the island and recorded many valuable observations on the rats before and during their disappearance. Christmas Island was evidently uninhabited by humans until the last quarter of the 19th century. With the discovery of phosphate deposits on the island, its exploitation became inevitable, and Christmas Island was formally annexed by the United Kingdom in 1888. An immediate result was increased ship traffic, with the result that in 1899 black rats (*Rattus rattus*) were inadvertently introduced. Within 5 years, the two endemic murids, the Christmas Island rat (*Rattus macleari*) and the bulldog rat (*Rattus nativitatis*), were seriously affected; populations declined precipitously, and individuals were described as behaving abnormally (e.g., nocturnally active rodents appearing during the daytime) and displaying evidence of infection with parasitic trypanosomiasis [48]. By 1908 it was believed that the native rats had become extinct, but that some of the endemic diversity should have survived in the form of *R. rattus *× *R. macleari* and *R. rattus *× *R. macleari* hybrids (as deduced from pelt characteristics). Samples of hybrids, together with earlier collections of the Christmas Island rat and the less abundant bulldog rat, have been stored in museum collections in the UK since then.

Wyatt et al. [10] extracted DNA from all available remaining Christmas Island and bulldog rats along with putative hybrid animals and black rats collected at the same time. Mitochondrial, nuclear, and trypanosome DNAs were amplified from the samples. Mitochondrial and nuclear DNA analysis clearly indicated *Rattus rattus* and *Rattus macleari* were biologically distinct, but that the alleged hybrids were in fact exclusively black rat morphotypes. Thus, the Christmas Island rat is completely extinct and its genetic endowment has not persisted in any form. Genetic evidence of the murid-specific trypanosome *Trypanosoma lewisi* was found in the true black rat and the Christmas Island rat samples. In samples of the bulldog rat, all collected earlier than 1899, no evidence of trypanosome infection could be found. Thus, detection of trypanosomes correlates with the arrival of invasive black rats and the subsequent extinction of the native rat species on Christmas Island (only inferential in the case of the bulldog rat). 

Like many other islands, Christmas Island is notable for having suffered mammal extinctions that cannot be explained by hunting pressure or sudden changes in climate [49]. The only other native ground-dwelling mammal on the island, the Christmas Island shrew (*Crocidura trichura*), survived many decades after the disappearance of the endemic rodents, but it has not been seen since 1985 despite considerable surveying effort [50]. More recently, the Christmas Island pipistrelle (*Pipistrellus murrayi*) appears to have become extinct in 2010, following an unexplained precipitous decline starting in the 1980s [50, 51]. The last remaining native mammal is the Christmas Island flying fox (*Pteopus melanotus natalis*), also declining rapidly. Whether these extinctions and declines subsequent to the loss of the endemic rats are disease-related might be resolved if appropriate samples were collected historically, although this possibility has not yet been investigated.

 In summary, and regardless of final cause, the rodent extinctions on Christmas Island display two interesting features. First, the extinctions were extremely rapid: the interval from first notice of decline to declared extinction was 10 years, with the actual event probably occurring within an even shorter period [52]. Second, the species affected would not normally be regarded as being particularly susceptible to extinction; murid rodents tend to be extremely adaptable, with high reproductive rates, and thus would normally be well buffered against population collapses sufficient to cause complete loss. The susceptibility factor at work in these cases was almost certainly long isolation on, and restriction to, a single small island. Among mammals the “island extinction effect” is particularly well investigated; more than 80% of the ~80–90 species of mammals that became extinct in the past 500 years were island dwelling [53]. Such losses are usually attributed to habitat destruction and the deleterious effects of invasive species, but under some circumstances introduced infectious diseases might be plausible as drivers.

There are a number of other, largely anecdotal, instances of mammalian species succumbing to unknown infectious diseases (see [19]). One example of considerable interest is the Tasmanian tiger or thylacine (*Thylacinus cynocephalus*). Although extensively hunted because of its supposed taste for sheep, until the turn of the 20th century thylacines were not uncommon. Thereafter they became exceedingly rare, with no confirmed kills logged after about 1910. Distemperlike outbreaks observed in captive thylacines, as well as widespread outbreaks in other Australian marsupials at the same time, have been taken as anecdotal evidence that disease played a role in the final disappearance of thylacines [1]. Validation of a role for disease in this extinction would be particularly difficult, as distemper viruses are single-stranded RNA paramyxoviruses that are unlikely to be preserved in museum skins or bone. 

## 7. Recent Cases of Infectious Disease Causing Serious Endangerment in Mammals

Several excellent review articles have analyzed the impacts of infectious disease on extant natural populations [1, 3, 4]. To these may be added several more examples of verified associations of disease and severe population decline in mammal species living in diverse environments. Whether any of these taxa will actually disappear is unknown at present, but the very fact that new examples are being discovered at a much higher rate than in past decades presumably indicates that infectious diseases have been undervalued as a significant cause of species endangerment. 

### 7.1. Koalas

Koalas are under siege by two major pathogens. Kola retrovirus (KoRV), frequently observed in captive koalas, is oncogenic and causes substantial mortality [54]. The virus is not distributed evenly across Australia, as its frequency within populations decreases from north to south. Given the history of overhunting of koalas in the last 120 years, especially in southern Australia, it was originally concluded that KoRV must have entered Australia within the last 200 years, travelling from north to south [55]. However, a recent study [14] utilizing historical samples demonstrated that KoRV was already widespread by the late 1800s in northern Australia. Furthermore, its evolution is very slow, which is consistent with the retrovirus having entered koala populations long before this and likely having caused disease throughout this time.

The second pathogen decimating koala populations at present is a specific strain of infectious *Chlamydia pecorum*. Infection can result in subsequent sterility or blindness [56]. There is some evidence that KoRV infection correlates with chlamydial infection [57]. This is plausible, as KoRV belongs to the gammaretrovirus family, many members of which are immunosuppressive. Infection with KoRV would thus provide opportunities for secondary infectious agents such as *Chlamydia* in less-resistant hosts. Koala populations exhibit low diversity in their mitochondrial DNA [58]; this lack of genetic diversity, if it affects overall resistance, may be emblematic of their inability to fend off infectious agents. According to the World Wildlife Foundation [59], serious overlapping infections of this kind could lead to the local extirpation, if not the outright extinction, of koalas within the next 50 years.

### 7.2. Myotis Bats

A cryophilous fungus (*Geomyces destructans*), introduced to the northeastern USA from Europe within the last decade, has been conclusively shown to cause massive mortality (“white nose syndrome”) in the little brown bat (*Myotis lucifugus*) [60]. Fungal infections do not normally induce severe disease in mammals, except in cases of immunosuppression (often itself caused by another pathogen). Interestingly, *Myotis myotis*, a closely related species endemic to Europe, is also infected with this fungus, but other than the characteristic “white nose” produced by fungal growth, animals suffer no negative symptoms and there is no increased mortality associated with infection [61–63]. *Geomyces destructans*, which only grows at cold temperatures, interrupts bat hibernation; this causes wintering animals to awaken more frequently than normal, seriously impairing their energy and water balances and ultimately leading to death. Although why these two species differ so dramatically in their response to infection is obscure, the consequences are clear; millions of little brown bats, including entire populations of some hibernacula, have died as a result of their incapacity to resist infection [61]. Indeed, current mortality rates suggest that the little brown bat may eventually suffer complete regional extinction [64].

### 7.3. Tasmanian Devils

Devil facial tumor disease (DFTD), a severe threat to the Tasmanian devil (*Sarcophilus harrisii*), is currently receiving a great deal of attention because of its highly unusual nature [65]. Unlike most infectious diseases, the agent in this case is not viral, bacterial, parasitic, or prion-derived (infectious protein). Rather, the agent is a tumor that is spread from individual to individual by mechanical transmission of tumor cells during agonistic encounters, which often involve aggressive behavior. The only similarly infectious cancer identified to date is canine transmissible venereal tumor (CTVT), which affects dogs. The DFTD source tumor evidently derived from multiple individual clones developed in a single female; male and female devils are equally susceptible, and the disease has now spread throughout Tasmania. Museum samples predating 1996 display no evidence of the disease, making it likely that DFTD is newly emergent. At the same time, there are clearly complex evolutionary dynamics at play; for example, different clonal lineages derived from the original source tumor vary in frequency, geographical distribution, and diversity [65]. How such tumors manage to escape the devil's immune system is unknown, but the ongoing threat is very real. Unless tumor-free populations can be established and protected, further population collapse, if not complete extinction, will be the probable outcome [66, 67].

## 8. Conclusions

Although host-pathogen interactions have been a subject of interest in conservation biology for some time, the possibility that disease might actually drive extinctions in certain contexts has rarely been considered. This is partly due to a general lack of knowledge concerning wildlife pathogens and their microbiology, but it also stems from a lack of well-researched and unequivocal examples of disease-induced loss of naturally occurring populations or species. We anticipate that, with the advent of endeavors such as the Human Microbiome Project [68] and the further development of next-generation sequencing, we will have an increasingly better understanding of microbiological processes in wildlife. Because of their relevance to human health, bat and rodent viromes are being explored with special intensity using high-throughput approaches, with the result that many novel—and potentially significantly pathogenic—viral strains have been identified in recent years [8, 69–72]. As such surveys increase, and sequencing costs decrease, we can expect to see a wealth of new data concerning microbiological diversity in wildlife, as well as new understandings of the natural history of host-pathogen relationships.

New insights will also come from archives that we have never heretofore been able to tap. It is already the case that entire genomes from historical samples (aboriginal human genome) or now-extinct species (woolly mammoths, neandertals) have proven amenable to reconstruction using modern methodologies; it may be confidently predicted that our ability to undertake such investigations will only improve in future [73]. With regard to ancient pathogens, investigators have already made exciting discoveries, such as the recent identification of previously undocumented strains of *Yersinia pestis* retrieved from 13th century plague pits [16]. At present, the most promising methods are based on either microarray or solution-based hybrid capture methods, in which a sequence of interest is either synthesized directly or produced by PCR, biotinylated, and then used to “capture” target sequences in samples from which sequencing libraries have been generated [74]. With DNA microarrays, multiple targets can be collected in a single experiment; when coupled with next-generation sequencing, extraordinary amounts of sequence information can be recovered at minimal cost. Although such methods have been primarily applied to modern DNA, there have been some notable successes with ancient DNA applications [75, 76]. Again, it is surely reasonable to assume that, as methodologies improve and are applied to fresh questions, we will be able to recover sequence data on ancient DNA-based pathogens at levels that would have been unimaginable only a decade or so ago. To be sure, RNA-based pathogens may remain resistant to study due to the poor preservation of RNA genomes after mortem, but even here there is reason to be optimistic. For example, improvements in protein sequencing may provide information on the identity of RNA-based viruses (either as such, or in the form of presence/absence information), even though the level of analytical detail will likely be persistently poorer than we can now achieve with DNA-genome-based pathogens [77].

Whether epizootics in wildlife occupying pristine environments are in fact increasing is a question that can only be settled by much larger pools of data than we have at present. Nevertheless, we believe that understanding the role of disease in provoking endangerment and extinction is critically important to the education of conservation professionals, if only because the contribution of disease to declines and outright extinction has likely been underestimated. What we do not understand, or ignore, may be what hurts us most.
